# ACE2 polymorphisms associated with cardiovascular risk in Uygurs with type 2 diabetes mellitus

**DOI:** 10.1186/s12933-018-0771-3

**Published:** 2018-09-18

**Authors:** Cheng Liu, Yanfang Li, Tianwang Guan, Yanxian Lai, Yan Shen, Abudurexiti Zeyaweiding, Haiyan Zhao, Fang Li, Tutiguli Maimaiti

**Affiliations:** 1 Department of Cardiology, Guangzhou First People’s Hospital, Medical School, South China University of Technology, #1 Panfu Road, Guangzhou, 510180 China; 2 Department of Cardiology, Guangzhou First People’s Hospital, Guangzhou Medical University, Guangzhou, 510180 China; 3 Department of Cardiology, Shufu People’s Hospital, Kashgar Region, Xinjiang Uygur Autonomous Region (XUAR) 844100 China

**Keywords:** Association, ACE2 polymorphism, Cardiovascular risk, Type 2 diabetes mellitus, Uygur

## Abstract

**Background:**

Type 2 diabetes mellitus (T2D), rapidly increasing to epidemic proportions, globally escalates cardiovascular disease risk. Although intensive interventions and comprehensive management of environmental risks factors for T2D are associated with reduced cardiovascular disease, such approaches are limited for individuals with high genetic T2D risk. In this study we investigated possible associations of ACE2 polymorphisms and cardiovascular risks in Uygur patients with T2D.

**Methods:**

275 Uygur T2D patients and 272 non-diabetic Uygur individuals were enrolled as study participants. 14 ACE2 polymorphisms were genotyped by Matrix-assisted laser desorption ionization time-of-flight mass spectrometry.

**Results:**

ACE2 SNP rs1978124, rs2048683, rs2074192, rs233575, rs4240157, rs4646156, rs4646188 and rs879922 were associated with T2D (all P < 0.05). The 8 diabetic risk related ACE2 SNPs were further associated with diabetic related cardiovascular complications or events but exhibited heterogeneity as fellows: firstly, almost all diabetic risk related ACE2 SNPs (all P < 0.05) were associated with increased SBP except rs1978124 and rs2074192, while rs2074192, rs4646188 and rs879922 were associated elevated DBP (all P < 0.05). Secondly, SNP rs4646188 was not correlated with any type of dyslipidemia (TRIG, HDL-C, LDL-C or CHOL), and the other 7 diabetic risk related loci were at least correlated with one type of dyslipidemia (all P < 0.05). In particular, rs879922 were simultaneously correlated with four type of dyslipidemia (all P < 0.05). Thirdly, ACE2 SNP rs2074192 and rs879922 were associated with carotid arteriosclerosis stenosis (CAS) ≥ 50% (both P < 0.05). Fourthly, ACE2 SNP rs2074192, rs4240157, rs4646188 and 879922 were associated with increased MAU (all P < 0.05). In addition, ACE2 SNP rs2048683, rs4240157, rs4646156, rs4646188 and rs879922 were linked to heavier LVMI (all P < 0.05), but only rs4240157, rs4646156 and rs4646188 were associated with lower LVEF (all P < 0.05).

**Conclusion:**

ACE2 SNP rs879922 may be a common genetic loci and optimal genetic susceptibility marker for T2D and T2D related cardiovascular risks in Uygurs.

**Electronic supplementary material:**

The online version of this article (10.1186/s12933-018-0771-3) contains supplementary material, which is available to authorized users.

## Background

Type 2 diabetes mellitus (T2D) is a clinical syndrome characterized by increased blood glucose, attributable to both genetic and environmental risk factors. T2D is one of the most serious challenges to global public health [1]. Cardiovascular disease is recognized as the leading cause in all-cause mortality of T2D, and the earlier onset of T2D, the more complication (e.g., increased blood pressure, dyslipidemia, arteriosclerosis cardiovascular disease (ASCVD), heart failure (HF), etc.) [2], the higher risk of cardiovascular death [3]. Thus a major goal of T2D prevention and control is to reduce the risk of cardiovascular events. In China the latest overall prevalence of T2D among adults is 10.9%, the age of onset is the trend of younger, and the number of T2D patients has been ranked the first in the world [4]. Under the circumstances, addressing the challenge of preventing and treating T2D in China should focus on decreasing cardiovascular events by intensive interventions and comprehensive management of cardiovascular risk factors in diabetic patients [5]. Although comprehensive management of those multiple modifiable risk factors (e.g., unhealthy diet, lack of physical activity, smoking, obesity, and dyslipidemia, etc.) is significant associated with lower blood sugar, lower blood pressure, lower LDL-C and lower incidence of T2D related cardiovascular events (e.g., ASCVD) [6], the cardiovascular benefits could be weaken or offset by metabolic memory effect from long-term elevated blood sugar and high genetic risk because it is impossible to modify the genetic structure [7]. Genetic background notwithstanding, recent studies examining the genetic basis of diabetes risk have revealed overall genetic risk factors and genetic loci leading to beta cell dysfunction, the major cause of T2D in China [8]. Thus, early identification (or) screening populations at high risk of T2D and T2D related cardiovascular complications, especially early-onset T2D, could provide a possible strategy for early prevention of diabetes.

Numerous candidate genes have been thus far been implicated in susceptibility to T2D, but few studies have focused on genes related to the renin-angiotensin-aldosterone system (RAAS). Angiotensin converting enzyme 2 (ACE2) is a key RAAS enzyme and a recently recognized target for the prevention and treatment of T2D. The gene encoding ACE2 maps to chromosome Xp22, spans 39.98 kbp of genomic DNA and consists of 20 introns and 18 exons. The ACE2 gene encodes a type I membrane-bound glycoprotein of 805 amino acids. Functional domains include a C-terminal transmembrane anchoring region, an N-terminal signal peptide and an HEXXH zinc binding metalloprotease motif. The ACE2 gene exhibits a high degree of genetic polymorphism and some polymorphisms are associated with T2D. There is also a high degree of genetic heterogeneity among ACE2 polymorphisms linked to T2D and not all variants exhibit association with T2D risk. Among Europeans, single nucleotide polymorphisms (SNPs) were not associated with T2D or T2D with diabetic nephropathy among persons of British (rs1978124, rs2074192, rs4646188 and rs2023802) or Finnish (rs2285666, rs2048684, rs879922, rs714205 and rs5978731) descent [9, 10]. Among persons of Australian descent rs2074192, rs4240157 and rs4646188 exhibited higher T2D with hypertension risk, rs1978124 was associated with risk of T2D related left ventricular remodeling [11].

However, the association of ACE2 SNPs with T2D and T2D related cardiovascular complications (e.g., hypertension and dyslipidemia) or events (e.g., ASCVD) in Chinese population are rarely reported. Theoretically, there may be common genetic basis between them [8, 12, 13] manifesting the characteristics of ethnic-specific genetic pleiotropy among cardio-metabolic traits [14]. In this study we investigated possible associations between ACE2 gene variation and cardiovascular risk in Uygur patients with type 2 diabetes mellitus.

## Materials and methods

### Study participants

This study was reviewed and approved by the Ethics Committee of Guangzhou First People’s Hospital, South China University of Technology. A total of 275 consecutive patients with T2D and 272 non-diabetes subjects from the south Xinjiang region were enrolled in the study from 2012 to 2017. All participants were long resident in the region and were from multi-generation resident families. All participants shared the same high- salt, sugar and fat diet. The newly T2D patients were diagnosed according to the criteria of the American Diabetic Association (ADA) guidelines of 1997 or World Health Organization (WHO) National diabetic group criteria of 2006 as follows: a single raised glucose reading with symptoms (polyuria, polydipsia, polyphagia and weight loss), otherwise raised values on two occasions, of either fasting plasma glucose (FPG) 7.0 mmol/L or with an oral glucose tolerance test (OGTT), 2 h after the oral dose a plasma glucose 11.1 mmol/L [15]. Blood pressure was measured in the seated position after 10 min of rest using a mercury sphygmomanometer by experienced and certified examiners, and measured in the brachial artery 3 times at 5-min intervals in at least two separate visits to the health care office. The mean of the last 2 measurements per visit was recorded as representative of clinic BP. All biochemistry tests were performed by standard methods in the Chemical Laboratory. Bilateral carotid and cardiac ultrasonic scanning was performed on admission to the study according to the measurement of degree of stenosis used in the North American Symptomatic Carotid Endarterectomy Trial [16] and the recommendations for chamber quantification from the American Society of Echocardiography [17], respectively.

### Genotyping assay

Genomic DNA was extracted from whole blood using the Maxwell RSC Whole blood DNA kit (Promega, Madison, WI), quantified using NanoDrop-1000 (ThermoFisher, Waltham, MA) and diluted to 10 ng/μL concentration. 14 ACE2 SNPs (rs1978124, rs2048683, rs2074192, rs2235306, rs2285666, rs233575, rs4240157, rs4646142, rs4646155, rs4646156, rs4646188, rs4830542, rs6632677 and rs879922) were identified based on existing literature and human genome sequence databases. Primers for ACE2 SNPs were designed based upon sequence information from GenBank using Primer 5.0 (Whitehead Institute Cambridge, Massachusetts, USA) and Operon’s Oligo software 7.60 (OperonTechnologies Inc., Alameda, California, USA). Primers are shown in Additional file 1: Table S1. ACE2 SNPs were analyzed using the Sequenom MassARRAY system according to previously described methods [18]. Genotyping accuracy was determined by genotype concordance between duplicate samples and was 100% for each SNP.

### Statistical analysis

The Hardy–Weinberg equilibrium was assessed for the control (non-diabetic) participants as shown in Additional file 1: Table S2. Analysis was performed using SPSS version 20 (SPSS, Chicago, IL) and PASS version 15 (Statistical Solution Ltd, Cork, Ireland). Categorical variables (nationality, gender, smoking, drinking, T2D and T2D complicated with CAS ≥ 50% were presented as frequencies. The relationship between each ACE2 SNP and those categorical variables were assessed using the Chi square test. The Odds ratio (OR) between control genotype and high T2D risk genotype for each ACE2 SNP among categorical variables was evaluated using binary logistic regression. Considering the possible false positive risk to the final result, Bonferroni adjustment was applied to adjust the *P* value obtained in multi-logit regression. Continuous variables (age, systolic blood pressure (SBP), diastolic blood pressure (DBP), body mass index (BMI), blood biochemical index, left ventricular mass index (LVMI) and left ventricular ejection fraction (LVEF)) were presented as mean ± SD. Significant differences for continuous variables were analyzed by two way ANOVA, One way ANOVA or independent-sample t-test according to our research design. The least significant difference (LSD) test was further used to assess differences for two subgroups after variance analysis, to show distinct differences with homogeneous variance, while the Games-Howell test was used for heterogeneous variance. A P value less than 0.05 was considered statistically significant. All probabilities are two-tailed.

## Result

### Characteristics of the study participants

Among Uygur participants, diabetic and non-diabetic subjects showed significant differences in SBP, DBP, BMI, triglyceride (TRIG), high-density lipoprotein cholesterol (HDL-C), Lipoprotein A, FBG, glycosylated hemoglobin (HbA1C), blood uric acid (UA), blood electrolytes (sodium and potassium), microalbuminuria (MAU), left heart remodeling (LVMI and LVEF) and the activation of RAAS (renin and Angiotensin I/II (ANG I/II)) (all P < 0.05) but not in gender, age, smoking, drinking, DBP, total cholesterol (CHOL), low-density lipoprotein cholesterol (LDL-C), lipoprotein A, the ratio of ApoA1/Apo B, renal function (Cr, BUN), liver function (ALT, AST, Alb) and high-sensitivity C-reactive protein (P > 0.05) (see Additional file 1: Table S3).

### Association of ACE2 SNPs and T2D

As shown in Table 1, ACE2 SNPs rs1978124 (P < 0.001), rs2048683 (P < 0.001), rs2074192 (P < 0.001), rs233575 (P < 0.001), rs4240157 (P < 0.001), rs4646156 (P < 0.001), rs4646188 (P < 0.001) and rs879922 (P = 0.005) were significantly associated with T2D except rs2235306, rs2285666, rs4646142, rs4830542 and rs6632677 (all P > 0.05, see Additional file 1: Table S4).

**Table 1:** Association of ACE2 SNPs with T2D in study participants

ACE2 SNPs		Non-diabetic (N/%)	Diabetic (N/%)	OR (95% CI)^a^	*P*-value
*rs1978124*					
*CC*		194 (71.3)	137 (49.8)	1.00	
*TT *+ *CT*		78 (28.7)	138 (50.2)	2.24 (1.53–3.26)	< 0.001
*rs2048683*					
*GG*		222 (81.6)	171 (62.2)	1.00	
*TT *+ *GT*		50 (18.4)	104 (37.8)	3.07 (2.02–4.68)	< 0.001
*rs2074192*					
*CC*		104 (38.2)	136 (49.5)	2.12 (1.43–3.13)	< 0.001
*TT *+ *CT*		168 (61.8)	139 (50.5)	1.00	
*rs233575*					
*CC *+ *CT*		58 (21.3)	112 (40.7)	3.00 (1.99–4.51)	< 0.001
*TT*		214 (78.7)	163 (59.3)	1.00	
*rs4240157*					
*CC *+ *CT*		64 (23.5)	124 (45.1)	2.75 (1.87–4.04)	< 0.001
*TT*		208 (76.5)	151 (54.9)	1.00	
*rs4646156*					
*AA *+ *AT*		54 (19.9)	104 (37.8)	2.71 (1.79–4.10)	< 0.001
*TT*		218 (80.1)	171 (62.2)	1.00	
*rs4646188*					
*CC *+ *CT*		102 (37.5)	64 (23.3)	1.00	
*TT*		170 (62.5)	211 (76.7)	2.18 (1.47–3.22)	< 0.001
*rs879922*					
*CC *+ *CG*		128 (47.1)	176 (64.0)	1.78 (1.19–2.65)	0.005
*GG*		144 (52.9)	271 (36.0)	1.00	

^a^After adjustment for nationality, gender, age, smoking and BMI

### Association of T2D risk related ACE2 SNPs with elevated blood pressure

**Fig. 1:**
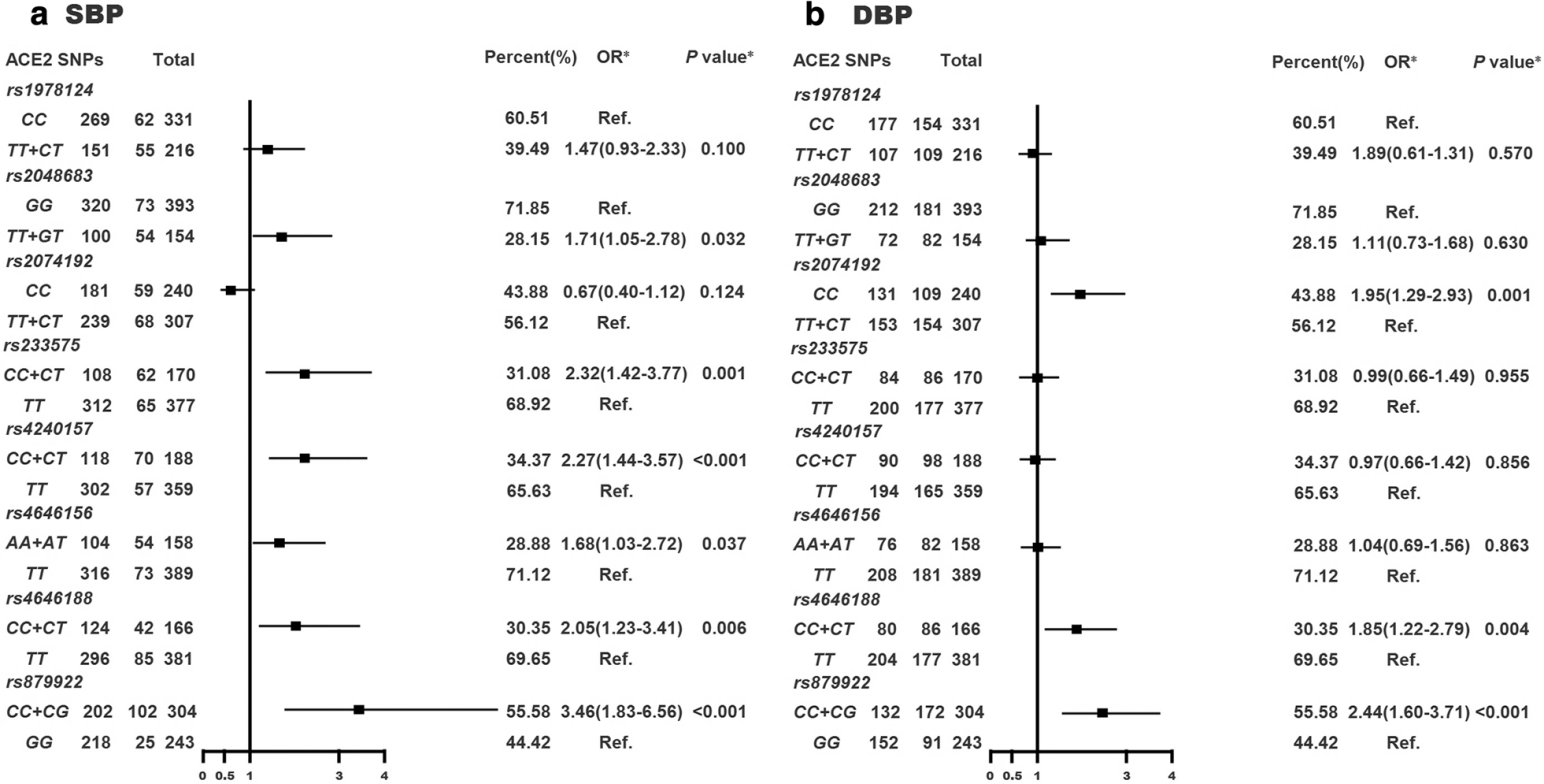
Association of T2D risk related ACE2 SNPs with elevated systolic (**a**) and diastolic (**b**) blood pressure in study participants. *After adjustment for gender, age, smoking, BMI and T2D

As shown in Fig. 1, T2D related ACE2 SNP rs4646188 and rs879922 were associated with increased SBP (P = 0.006 and < 0.001) and DBP (P = 0.004 and < 0.001) while rs1978124 was not (both P > 0.05). rs2048683 (P = 0.032), rs233575 (P = 0.001), rs4240157 (P < 0.001) and rs4646156 (P = 0.037) were only correlated with increased SBP while rs2074192 (P = 0.001) was only correlated with elevated DBP.

### Association of T2D risk related ACE2 SNPs with dyslipidemia

**Fig. 2:**
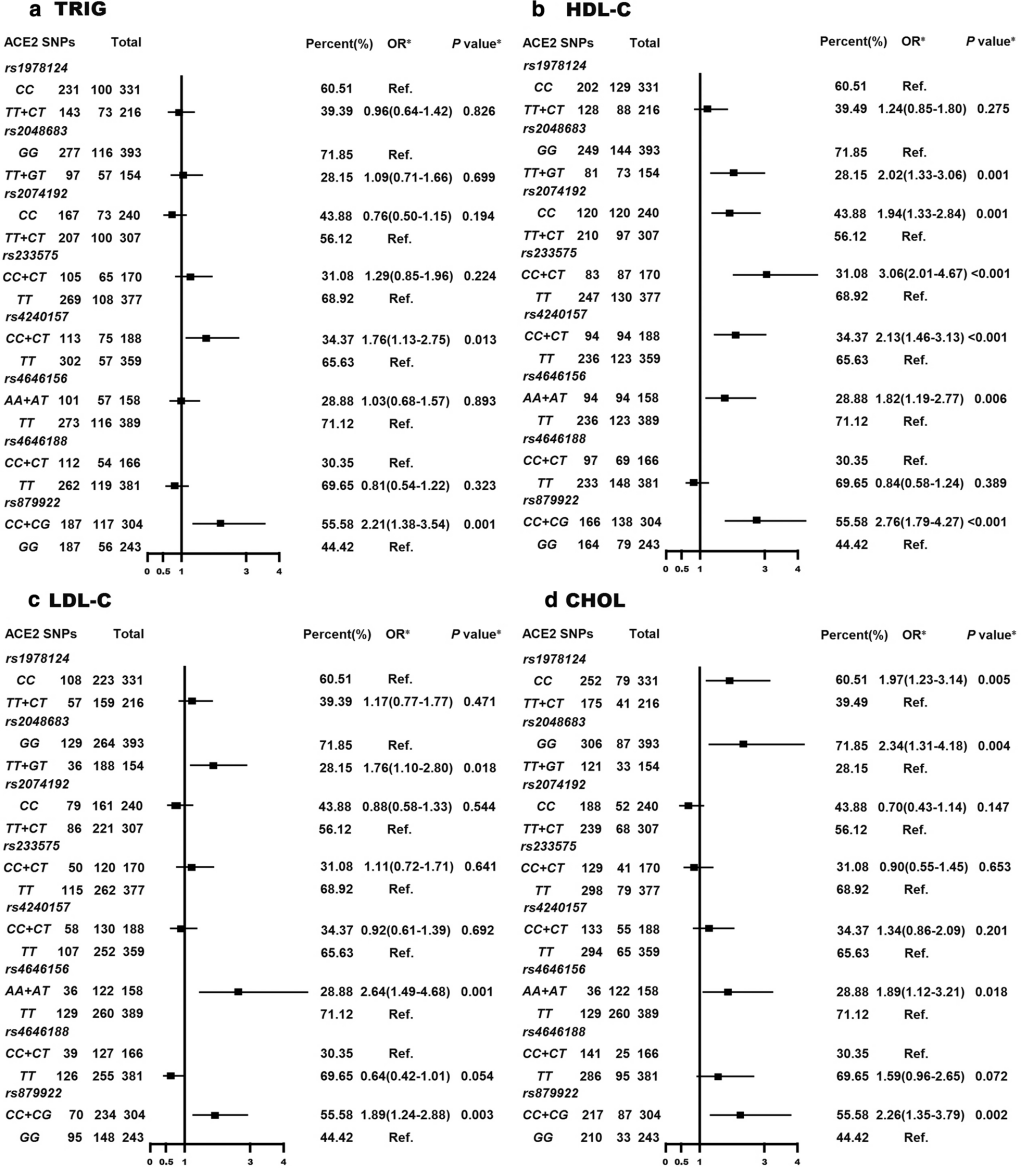
Association of T2D risk related ACE2 SNPs with dyslipidemia in study participants. **a** TRIG; **b** HDL-C; **c** LDL-C; **d** CHOL. *After adjustment for gender, age, BMI and T2D

As shown in Fig. 2, T2D risk related ACE2 SNP rs1978124 and rs4646188 were not correlated with any type of dyslipidemia (TRIG, HDL-C, LDL-C, CHOL), rs1978124, rs2074192 and rs233575 were correlated with one type of dyslipidemia (CHOL or HDL-C, all P < 0.05), rs4240157 was correlated with two type of dyslipidemia (TRIG and HDL-C, both P < 0.05), rs2058683 and rs4646156 were correlated with three type of dyslipidemia (HDL-C, LDL-C and CHOL, all P < 0.05) and rs879922 correlated with four type of dyslipidemia (TRIG, HDL-C, LDL-C and CHOL, all P < 0.05).

### Association of T2D risk related ACE2 SNPs with CAS ≥ 50%

As shown in Table 2, ACE2 SNPs rs2074192 (P = 0.045) and rs879922 (P = 0.022) were associated with T2D complicated by CAS ≥ 50%.

**Table 2:** Association of T2D risk related ACE2 SNPs with CAS ≥ 50% in study participants

ACE2 SNPs		Non-CAS ≥ 50% (N/%)	CAS ≥ 50% (N/%)	OR (95% CI)*	*P*-value*
*rs2074192*					
*CC*		190 (40.4)	50 (64.9)	1.97 (1.01–3.82)	0.045
*TT *+ *CT*		280 (59.6)	27 (35.1)	1.00	
*rs879922*					
*CC *+ *CG*		244 (51.9)	60 (77.9)	2.30 (1.12–4.68)	0.022
*GG*		226 (48.1)	17 (22.1)	1.00	

*After adjustment for T2D, smoking, age, BMI, DBP, LDL-C and Ang II

### Association of T2D risk related ACE2 SNPs with MAU

As shown in Table 3, T2D patients with the high diabetic risk genotype of rs2074192 (CC, P = 0.038), rs4240157 (CC + CT, P < 0.001), rs4646188 (TT, P < 0.001) and rs879922 (CC + CG, P = 0.049) were associated with increased MAU level.

**Table 3:** Association of T2D risk related ACE2 SNPs and MAU in study participants

ACE2 SNPs		ACR (mg/g)		
		Non-diabetic	Diabetic	*P*-*value*
*rs2074192*				
*CC*		90.2 ± 28.6	98.7 ± 26.5	0.019
*TT *+ *CT*		87.5 ± 22.1	92.3 ± 26.7	0.088
*P*-*value*		0.404	0.048	
*rs4240157*				
*CC *+ *CT*		85.8 ± 25.6	104.7 ± 29.7	< 0.001
* TT*		89.3 ± 24.5	87.9 ± 21.2	0.552
*P*-*value*		0.324	< 0.001	
*rs4646188*				
*CC *+ *CT*		85.3 ± 23.1	78.8 ± 18.7	0.059
*TT*		90.4 ± 25.6	100.5 ± 26.7	< 0.001
*P*-*value*		0.100	< 0.001	
*rs879922*				
*CC *+ *CG*		85.9 ± 22.6	100.7 ± 29.7	< 0.001
*GG*		90.9 ± 26.4	86.2 ± 16.8	0.089
*P*-*value*		0.091	< 0.001	

*ACR* urinary albumin-to-creatinine ratio

### Association of T2D risk related ACE2 SNPs with left heart remodeling

As shown in Table 4, T2D patients with the high diabetic risk genotype of rs2048683 (TT + CT, P = 0.007), rs4240157 (CC + CT, P = 0.008), rs4646156 (AA + AT, P = 0.003), rs4646188 (TT, P = 0.010) and rs879922 (CC + CG, P < 0.001) were linked to heavier LVMI, but only rs4240157 (P = 0.020), rs4646156 (P = 0.043), rs4646188 (P = 0.018) were further associated with lower LVEF.

**Table 4:** Association of T2D risk related ACE2 SNPs with left heart remodeling

ACE2 SNPs		LVMI (g/m^2^)			LVEF (%)		
		Non-diabetic	Diabetic	*P*-*value*	Non-diabetic	Diabetic	*P*-*value*
*rs1978124*							
*CC*		86.5 ± 14.8	92.3 ± 15.4	0.001	62.3 ± 5.9	58.6 ± 7.6	< 0.001
*TT *+ *CT*		88.5 ± 16.7	95.2 ± 17.3	0.006	61.7 ± 7.3	57.7 ± 8.0	< 0.001
*P*-*value*		0.347	0.142		0.579	0.336	
*rs2048683*							
*GG*		86.6 ± 14.5	91.9 ± 16.2	0.001	62.0 ± 6.0	59.0 ± 7.3	< 0.001
*TT *+ *GT*		89.4 ± 18.6	96.8 ± 16.3	0.013	62.5 ± 7.7	56.7 ± 8.4	< 0.001
*P*-*value*		0.310	0.015		0.698	0.020	
*rs2074192*							
*CC*		85.8 ± 15.4	93.3 ± 13.1	< 0.001	62.4 ± 6.1	57.7 ± 7.0	< 0.001
*TT *+ *CT*		87.9 ± 15.3	94.2 ± 19.1	0.002	62.0 ± 6.5	58.5 ± 8.5	< 0.001
*P*-*value*		0.273	0.652		0.627	0.412	
*rs233575*							
*CC *+ *CT*		85.9 ± 14.1	96.8 ± 15.3	< 0.001	62.8 ± 7.0	56.9 ± 8.3	< 0.001
*TT*		87.4 ± 15.7	91.7 ± 16.8	0.012	61.9 ± 6.1	58.9 ± 7.4	< 0.001
*P*-*value*		0.513	0.011		0.382	0.041	
*rs4240157*							
*CC *+ *CT*		86.6 ± 13.9	97.7 ± 16.1	< 0.001	62.4 ± 6.6	56.5 ± 8.5	< 0.001
*TT*		87.2 ± 15.8	90.5 ± 15.9	0.054	62.0 ± 6.3	59.4 ± 7.0	< 0.001
*P*-*value*		0.766	< 0.001		0.698	0.003	
*rs4646156*							
*AA *+ *AT*		90.2 ± 18.1	96.8 ± 16.3	0.020	62.1 ± 7.5	56.7 ± 8.4	< 0.001
*TT*		86.3 ± 14.5	91.9 ± 16.2	< 0.001	62.1 ± 6.0	59.0 ± 7.3	< 0.001
*P*-*value*		0.152	0.015		0.967	0.020	
*rs4646188*							
*CC *+ *CT*		86.4 ± 16.6	88.1 ± 12.0	0.487	62.0 ± 6.1	61.1 ± 8.2	0.432
*TT*		87.5 ± 14.6	95.5 ± 17.1	< 0.001	62.2 ± 6.5	57.2 ± 7.5	< 0.001
*P*-*value*		0.575	< 0.001		0.798	0.001	
*rs879922*							
*CC *+ *CG*		89.0 ± 15.0	96.9 ± 16.9	< 0.001	62.2 ± 6.4	57.5 ± 8.9	< 0.001
*GG*		85.4 ± 15.5	88.2 ± 13.9	0.147	62.0 ± 6.3	59.3 ± 5.4	0.001
*P*-*value*		0.058	< 0.001		0.758	0.039	

## Discussion

T2D is rapidly developing into an epidemic and dramatically increases the global cardiovascular events that have become a serious public health problem especially in developing countries (e.g., China) [19]. During the last decade in China, the prevalence of T2D has continuously increased, and followed by an increased risk of cardiovascular morbidity and mortality, including hypertension, dyslipidemia, macrovascular (e.g. ASCVD) and microvascular (e.g. MAU) complications [20]. T2D prevalence also differs among different groups, differentially affecting Han and different non-Han populations [4], especially in the minority areas (e.g., Xinjiang region). The prevalence of T2D in the Uygur population of Xinjiang is particularly high in both urban and rural environments due to unhealthy lifestyle related factors (such as high BMI, unhealthy diet, and low physical activity) and an aging population [4], while at the same time diabetes related cardiovascular risk is extremely high [21]. Thus, early identification and assessing populations at high risk of T2D and T2D related cardiovascular diseases are the key steps in diabetes prevention and control.

### ACE2 gene polymorphisms and risk of diabetes

This study investigated possible associations between ACE2 polymorphisms and T2D in the Uygur population of the Xinjiang region of China. We found that patients carrying the genotypes of rs879922 (CC + CG) had a moderate risk to develop T2D while rs1978124 (TT + CT), rs2048683 (TT + GT), rs2074192 (CC), rs233575 (CC + CT), rs4240157 (CC + CT), rs4646156 (AA + AT) and rs4646188 (TT) had a high risk. This is the first report showing that SNP rs4830542 were not associated with T2D (see Additional file 1: Table S4). We also found that ACE2 SNP rs2285666 was not linked to T2D in Uygur participants, which is also true for persons of European descent [9]. Indeed, HbA1c is an important biological marker for predicting cardiovascular events in T2D patients [22]. We also found that the levels of HbA1C in T2D Uygur patients with the high diabetic risk genotypes of the three loci (rs2074192, rs4240157 and rs879922) increased significantly (see Additional file 1: Table S10), suggesting that those would be at higher potentially risk of cardiovascular events.

### ACE2 gene polymorphisms and diabetic related cardiovascular risk

Essential hypertension (EH) is recognized as the leading cause in global death of vascular disease [23]. Although the BP targets in diabetic hypertensive individuals are controversial, it was common practice to aim for BP targets lower than 130/80 mmHg in most diabetic patients [24], which will help reduce cardiovascular events (e.g., at least stroke) [39]. In this study we found that 7 diabetic risk related ACE2 SNPs were associated with increased SBP or DBP with exception of rs1978124. Our results are partial consistent with a study in a diabetic Australian Caucasian population which reported association of four ACE2 SNPs (rs4646188, rs4240157, rs2074192 and rs1978124) with hypertension. We found that rs4646188 was associated with high risk of SBP ≥130 mmHg and moderate risk of DBP ≥80 mmHg, rs4240157 and was only associated with high risk of increased SBP while rs2074192 was only associated with moderate risk of increased DBP. Framingham Study showed that a 1.5–2.0 fold increased risk of cardiovascular events when SBP increased from 130 mmHg to 139 mmHg [25]. These results suggest that those elevated SBP risk related loci may be genetic factors contributing to T2D related cardiovascular events risk in Uygurs.

Beside hypertension, dyslipidemia is another important risk factor for increased prevalence of cardiovascular events in T2D [23]. Our study found that abnormal blood lipid metabolism was significantly higher in diabetic patients compared to non-diabetic subjects and was characterized by high triglycerides and low HDL-C but normal levels of LDL-C and total cholesterol, which are consistent with previously reported characteristics of the lipid spectrum in the Chinese T2D patient population [26]. In previous studies SNP rs2285666 was not linked to dyslipidemia in T2D patients, but its relationship with various subtypes of dyslipidemia is not shown [27]. Especially, LDL-C is the most important risk factor for cardiovascular events (e.g., ASCVD) in T2D patients. Also, significantly increased cardiovascular events are associated with LDL-C levels above 1.8 mmol/L. To the best of our knowledge, this is the first more comprehensive study to investigate the association of ACE2 gene polymorphism with dyslipidemia in T2D patients. We found that 3 ACE2 SNPs (rs2048683, rs4646156 and rs879922) were correlated with increased risk of LDL-C ≥1.8 mmol/L in Uygur T2D patients, and rs879922 was significantly associated with four type of dyslipidemia. The high TRIG level and low HDL-C level are also powerful independent predictors of cardiovascular events independent of LDL-C levels [28]. ACE2 SNP rs4240157 and rs879922 were associated with high TRIG level, and almost all diabetic risk related ACE2 SNPs were associated with low HDL-C level except rs1978124 and rs4646188, but rs1978124 was correlations with high CHOL level as well as the other 3 SNPs (rs2048683, rs4646156 and rs879922). Remarkably, although there were no significant differences on LDL-C and CHOL levels between non-diabetic and diabetic individuals, but we found that T2D patients with the high diabetic risk or control genotypes of 4 diabetic risk related ACE2 SNPs (rs1978124, rs2048683, rs4646156 and rs879922) had a higher risk to develop high levels of LDL-C (≥ 1.8 mmol/L) and CHOL (≥ 5.2 mmol/L) adjusted for gender, age, BMI, T2D and ACE2 SNPs, which further suggests that the 4 ACE2 SNPs may be potential influential factors of dyslipidemia in diabetics. However, the mechanism behind this remains unclear, it is speculated that it may be related to a body-size dependent manner [29] and the BMI level was indeed statistically different between non-diabetic and diabetic between non-diabetic and diabetic individuals in our study. These results suggest that ACE2 SNPs correlations with elevated risk of dyslipidemia were obvious heterogeneity, and rs879922 was associated with four type of dyslipidemia suggesting it may be a genetic factor contributing to T2D with dyslipidemia in Uygurs.

Unfortunately, despite diabetics received standard hypoglycemic therapy, hypotensive therapy and lipid-lowering therapy, there is still a significant increase residual risk of macrovascular (e.g., ASCVD) and microvessels (e.g., retinopathy, MAU, etc.) complications, that is not only related to atherogenic dyslipidemia (high TRIG and low HDL-C level as described above) [30] but also at least partly related to the genetic background of individuals [31]. It’s well known that there is a common genetic basis for dyslipidemia and ASCVD [8]. Our results showed that 2 SNPs (rs2285666 and rs4646142) was not associated with T2D (see Additional file 1: Table S4) but exhibited association with T2D with moderate risk of atherogenic dyslipidemia (see Additional file 1: Table S6, S7), which were consistent with previously reported association of the loci with ASCVD (e.g., coronary heart disease [32], ischemic stroke [33]) in T2D patients as well as cardiovascular death in European females [34]. Similar effects also existed in between rs10911021 and CAD in T2D [35] as well as between CLOCK polymorphism and stroke in T2D patients [36]. According to the 2014 NLA recommendations [37], carotid arteriosclerosis stenosis (CAS) ≥ 50% is defined as a new type of ASCVD. In our study we newly found that both diabetic and atherogenic dyslipidemia risk related ACE2 SNPs (rs2074192 and rs879922) were respectively linked to moderate and high risk of CAS ≥ 50%, which previously reported that both loci had nothing to do with the recurrence risk of stroke [38] but were associated with sudden cardiac arrest [39] and retinopathy [40]. On the other hand, MAU is another key biomarker of microvascular complications in T2D and is one of the most valuable factors for predicting cardiovascular events in T2D [41]. We found that the 2 carotid arteriosclerosis risk related SNPs were also linked to increased MAU level besides rs4240157 and rs4646188. In addition, rs1978124 was correlations with elevated CHOL risk, and also exhibited association with death in European patients with acute coronary syndromes [42].

T2D is acknowledged as a key risk factor for atherosclerosis but is not yet fully recognized as an important independent risk factor for HF [43]. T2D complicated with HF is very common and associated with increased risk for all-cause and cardiovascular mortality and HF hospitalization. Nevertheless, diabetes associated HF, especially HF with preserved ejection fraction (HFpEF) frequently goes unrecognized. Importantly, there are as yet no effective treatment strategies to reduce all-cause death in patients with HFpEF, which highlights the importance of early identification of damage indices of HF in clinical practice [44]. LVMI and LVEF are important early damage biomarkers reflecting left heart remodeling. We found that 5 SNPs (rs2048683, rs4240157, rs4646156, rs4646188 and rs879922) were associated with increased LVMI and 3 SNPs (rs4240157, rs4646156 and rs4646188) were associated with lower LVEF. This is the first report showing that SNP rs2048683 and rs4646188 were associated with increased LVMI. With exception of rs233575, our findings coincides with the observation by Lieb et al. [45] who reported 4 ACE2 SNPs (rs4240157, rs4646156, rs879922 and rs233575) were associated with higher LVMI. However, our results are in contrast to the study by Sheila K et al. [11] who reported that rs1978124 was correlated with higher LVMI and lower LVEF while rs4646188 was not. These observations suggest that rs4646188 and rs879922 may be genetic susceptibility markers of early T2D related left heart remodeling in Uygurs.

It is well known that the activation of RAAS not only plays an important role in the occurrence and development of diabetes mellitus but also runs through the whole diabetic related cardiovascular event chain (e.g., hypertension, dyslipidemia, ASCVD, MAU and left ventricular remodeling, etc.) [46]. ACE2, a homolog of ACE, is a monocarboxypeptidase that converts angiotensin II (Ang II) into angiotensin 1–7 (Ang 1–7) which, by virtue of its actions on the Mas receptor, opposes the molecular and cellular effects of Ang II, and exhibites notable cardiovascular protective effects [47]. Although the roles of ACE2 gene polymorphisms (mutations or variants) on diabetes and other associated cardiovascular complications were incompletely understood, it may be related to the cross-talk between ACE2/Ang-(1–7)/Mas axis and ACE/Ang II/AT1 axis [48]. ACE2 gene polymorphisms (e.g., rs2106809 [49], rs2074192 [48]) were associated with downregulation of circulating Ang-(1–7). The deletion of ACE2 in mice model was associated with increased plasma and tissue Ang II levels [50], led to impaired glucose homeostasis [51] and cardiovascular damage [50]. The molecular mechanism might be involved in posttranscriptional regulation via microRNA, because it has been reported that the changes of ACE2 expression was in protein level rather than mRNA level in diabetic mice [52], and microRNA might regulate RAAS activity via by altering the interaction between microRNA and mRNA of targeted gene [49] that need to be investigated further.

Some limitations should be mentioned. First, since our sample size is not large enough, further prospective large sample studies are needed to validate our findings. Secondly, the possibility of false-positive findings should be considered especially for secondary study based on our results.

## Conclusion

ACE2 gene variants were associated with T2D and T2D with elevated blood pressure, dyslipidemia, carotid arteriosclerosis, MAU and left ventricular remodeling with characteristic of genetic pleiotropy in Uygurs. Our findings suggest that ACE2 SNPs rs879922 may be a common genetic loci and optimal genetic susceptibility marker for T2D and T2D related cardiovascular complications in Uygur. Our observations further support that the genetic predisposition of ACE2 SNPs associated with the risk of T2D and T2D related cardiovascular risk should need large-scale evaluation.

## Additional file

**Additional file 1.**

## Abbreviations

ACE2: angiotensin converting enzyme 2
ACR: urinary albumin-to-creatinine ratio
Alb: albumin
ALD: aldosterone
ALT: alanine aminotransferase
Ang I/II: angiotensin I/II
ASCVD: arteriosclerosis cardiovascular disease
AST: aspartate aminotransferase
BMI: body mass index
BUN: blood urea nitrogen
CAS: carotid arteriosclerosis stenosis
CHOL: hypercholesterolemia
Cr: creatinine
DBP: diastolic blood pressure
EH: essential hypertension
FBG: fasting blood glucose
HbA1C: glycosylated hemoglobin
HDL-C: high-density lipoprotein cholesterol
HsCRP: high-sensitivity C-reactive protein
LDL-C: low-density lipoprotein cholesterol
Lp(a): lipoprotein A
LVMI: left ventricular mass index
LVEF: left ventricular ejection fraction
MAU: microalbuminuria
RAAS: renin–angiotensin–aldosterone system
SBP: systolic blood pressure
SNP: single nucleotide polymorphism
T2D: type 2 diabetes mellitus
TRIG: hypertriglyceridemia
UA: blood uric acid
