# Supplementary material for: ACE2 polymorphisms associated with cardiovascular risk in Uygurs with type 2 diabetes mellitus
Source: Cardiovasc Diabetol. 2018 Sep 18;17:127. doi: 10.1186/s12933-018-0771-3 (PMC6142339; doi:10.1186/s12933-018-0771-3)
Supplement: Supplementary file 1 — Additional file 1. [file 12933_2018_771_MOESM1_ESM.docx]

**Additional file 1：Additional Method and Results**

**Additional Method**

**Carotid and cardiac ultrasonography**

Bilateral carotid and cardiac ultrasonic scanning was performed on admission to the study. The near and far walls of bilateral common carotid artery, bifurcations, and 1cm of the internal and external carotid arteries were scanned for the presence of carotid arteriosclerosis stenosis(CAS) ≥50% (recorded as the average of two independent experienced physician measurements according to the measurement of degree of stenosis used in the North American Symptomatic Carotid Endarterectomy Trial[1]) with a 3/9 MHz ultra-wideband linear array transducer (iU22, Philips, NL). Left ventricular mass index (LVMI) and left ventricular ejection fraction (LVEF) was measured using M-mode or two-dimensional echocardiography in the parasternal long-axis view at the end-ventricular systole with a 1.7/3.4 MHz linear array transducer (Vivid 7, GE Healthcare, USA) over 4 cardiac cycles according to recommendations for chamber quantification from the American Society of Echocardiography[2].

**Additional Results**

**ACE2 SNPs and genotype frequencies**

Not all ACE2 SNPs examined were in Hardy-Weinberg equilibrium (P>0.05), and heterogeneity was observed in study participants (see Additional Table S2). In non-diabetic participants only 7 ACE2 SNPs (rs1978124, rs2235306, rs4646155, rs4646156, rs4646188, rs6632677 and rs879922) were in Hardy-Weinberg equilibrium (P>0.05).

**Association of ACE2 SNPs and T2D**

As shown in Additional Table S4, ACE2 SNPs rs2235306, rs2285666, rs4646142, rs4830542 and rs6632677 were not associated with T2D (all P>0.05). In addition, significant differences were observed between diabetic and non-diabetic Uygur participants for genotype distributions of ACE2 SNPs rs4646155 (P=0.001), but considering the limit of the sample size, the estimation for the potential association between the loci and T2D may be biased. Therefor the OR for the rs4646155 will not be estimated.

**Association of T2D risk related ACE2 SNPs with elevated blood pressure**

As shown in in Additional Table S5, T2D related ACE2 SNP rs4646188 and rs879922 were associated with increased SBP (P=0.006 and <0.001) and DBP (P=0.004 and <0.001) while rs1978124 was not (both P>0.05). rs2048683 (P=0.032), rs233575 (P=0.001), rs4240157 (P<0.001) and rs4646156 (P=0.037) were only correlated with increased SBP while rs2074192 (P=0.001) was only correlated with DBP.

**Association of T2D risk related ACE2 SNPs with dyslipidemia**

As shown in Additional Table S6-S9, T2D risk related ACE2 SNP rs1978124 and rs4646188 were not correlated with any type of dyslipidemia (TRIG, HDL-C, LDL-C, CHOL), rs1978124, rs2074192 and rs233575 were correlated with one type of dyslipidemia (CHOL or HDL-C, all P<0.05), rs4240157 was correlated with two type of dyslipidemia(TRIG + HDL-C, both P<0.05), rs2058683 and rs4646156 were correlated with three type of dyslipidemia (HDL-C, LDL-C and CHOL, all P<0.05) and rs879922 correlated with four type of dyslipidemia (TRIG, HDL-C, LDL-C and CHOL, all P<0.05).

**The HbA1C level difference between different genotypes of T2D risk related ACE2 SNPs in study participants with or without T2D**

As shown in Additional Table S10, The HbA1C level of T2D patients with the high diabetic risk genotypes of 3 ACE2 SNPs (rs2074192, rs4240157 and rs879922) were not only significantly higher than that with the opposite genotypes (all P<0.05), but also obviously higher than the HbA1C level of non-diabetic participants with the same genotype (all P<0.05).

**Additional Table legends**

Additional Table S1 ACE2 SNP primers used in the Sequenom MassARRAY system

Additional Table S2 Descriptive information on ACE2 SNPs in study participants

Additional Table S3 Baseline characteristics of study participants

Additional Table S4 Association of ACE2 SNPs with T2D in study participants

Additional Table S5 Association of T2D risk related ACE2 SNPs with elevated blood pressure in study participants

Additional Table S6 Association of ACE2 SNPs with increased TRIG (≥1.7mmol/L) in study participants

Additional Table S7 Association of ACE2 SNPs with decreased HDL-C (<1.0mmol/L) in study participants

Additional Table S8 Association of T2D risk related ACE2 SNPs with increased LDL-C (≥1.8mmol/L) in study participants

Additional Table S9 Association of T2D risk related ACE2 SNPs with increased CHOL (≥5.2mmol/L) in study participants

Additional Table S10 The HbA1C level difference between different genotypes of T2D risk related ACE2 SNPs in study participants with or without T2D

Additional Table S1 The primers of ACE2 SNPs in the Sequenom MassARRAY system

| **NQ.** | **SNP_ID** | **2nd-** **reverse PCR primer (5’-3’)** | **1st-** **forward PCR primer (5’-3’)** | **extension primer (5’-3’)** |
| --- | --- | --- | --- | --- |
| 1 | *rs1978124* | *ACGTTGGATGGAGAGAACTTTGGAAACCTG* | *ACGTTGGATGAAGCTGCTGATGTAGAAGTG* | *CCATATCTCTATCTGATGGAC* |
| 2 | *rs2048683* | *ACGTTGGATGCAGTGAACATGGCAGTGTAG* | *ACGTTGGATGTGATCCAGCAATCCCTCTTC* | *GGCAGTGTAGATATCTTTATGAAG* |
| 3 | *rs2074192* | *ACGTTGGATGTTAGGTTCATCAACAGCTCC* | *ACGTTGGATGCCCTTAAACACAGCAGTCAC* | *CAAGGGTGGAAATGTATAAATGGTTGG* |
| 4 | *rs2235306* | *ACGTTGGATGAATAACAGTCTCTCTCCCCC* | *ACGTTGGATGTTCCTGGGTAGATGGCAATG* | *GTTTTCCTGCACACCATCTGAT* |
| 5 | *rs2285666* | *ACGTTGGATGCTGAGAGAAAAGTAAATTTCA* | *ACGTTGGATGCCAGATAATCCACAAGAATGC* | *CATAATCACTACTAAAAATTAGTAGC* |
| 6 | *rs233575* | *ACGTTGGATGAGGTCCTATGACCAAGTCTC* | *ACGTTGGATGTTTCTTATGTGCCTCCCCAG* | *TCCTATGACCAAGTCTCTATAGTA* |
| 7 | *rs4240157* | *ACGTTGGATGTTGCTCAGTGAATTGGCCTC* | *ACGTTGGATGTTTCCATGCAGTGAGGGTTG* | *CTCAGAACATTACAGAATCAAAC* |
| 8 | *rs4646142* | *ACGTTGGATGTTAGGTGAAGCGTTCCTGTC* | *ACGTTGGATGAGCCCATGGACCCAATAAAG* | *GGAGATGGTTGCTTTGTAGTCTC* |
| 9 | *rs4646155* | *ACGTTGGATGGGCATGTTCTTAACCTTGGC* | *ACGTTGGATGCCAATATGACCCTGTAAACC* | *GGATGAACCTTGGCAAAATAAACTT* |
| 10 | *rs4646156* | *ACGTTGGATGGGGAAATAGATATGATGGGC* | *ACGTTGGATGCCTTAGGTACTTGGACCTTC* | *GGGCCATGGAACAGG* |
| 11 | *rs4646188* | *ACGTTGGATGGATATTCTACTCAGAAACG* | *ACGTTGGATGCTCTGTGTTCCCTTCTGTTG* | *GGGGAACGTAGAATTTTAGTTGAATG* |
| 12 | *rs4830542* | *ACGTTGGATGAGGATGAGCTCATGCACAAG* | *ACGTTGGATGCAAAAATAAGAAGAAGAAAGG* | *TCTTTCTGGTCTCCCTCT* |
| 13 | *rs6632677* | *ACGTTGGATGAGCCTCAGAAGAGACCATAG* | *ACGTTGGATGAGTTCAGCTGGATCTTCTGC* | *CTCTACCATAGCTCTAGCCA* |
| 14 | *rs879922* | *ACGTTGGATGGCTCCAGCAAATTCAAGGAC* | *ACGTTGGATGGGCAGTTTATTGTACATTGTG* | *CTCAAGGACTGGGGTTA* |

Additional Table S2 Descriptive information on ACE2 SNPs in study participants

| **NQ** | **ACE2 SNPs** | **MAF in**  **CHB/CEU*** | **Major/minor allele** | **MAF** | | **Power** | ***P_HWE_*-value^#^** |
| --- | --- | --- | --- | --- | --- | --- | --- |
|  |  |  |  | **Non-diabetic** | **Diabetic** |  |  |
| 1 | *rs1978124* | 0.006/0.483 | *C/T* | 0.195 | 0.364 | 0.998 | 0.129 |
| 2 | *rs2048683* | 0.006/0.409 | *G/T* | 0.125 | 0.262 | 0.852 | 0.019 |
| 3 | *rs2074192* | 0.463/0.349 | *C/T* | 0.478 | 0.316 | 0.999 | 0.022 |
| 4 | *rs2235306* | 0.456/0.047 | *T/C* | 0.188 | 0.229 | 0.997 | 0.068 |
| 5 | *rs2285666* | 0.488/0.262 | *C/T* | 0.430 | 0.385 | 0.999 | 0.011 |
| 6 | *rs233575* | 0.006/0.362 | *T/C* | 0.136 | 0.269 | 0.915 | <0.001 |
| 7 | *rs4240157* | 0.044/0.383 | *T/C* | 0.162 | 0.327 | 0.981 | 0.033 |
| 8 | *rs4646142* | 0.488/0.261 | *G/C* | 0.430 | 0.400 | 0.999 | 0.011 |
| 9 | *rs4646155* | 0.038/0.000 | *T/C* | 0.022 | 0.087 | 0.402 | 0.383 |
| 10 | *rs4646156* | 0.006/0.403 | *T/A* | 0.129 | 0.255 | 0.878 | 0.155 |
| 11 | *rs4646188* | 0.000/0.087 | *T/C* | 0.301 | 0.160 | 0.999 | 0.202 |
| 12 | *rs4830542* | 0.044/0.383 | *T/C* | 0.243 | 0.320 | 0.999 | 0.008 |
| 13 | *rs6632677* | 0.094/0.007 | *G/C* | 0.051 | 0.080 | 0.050 | 0.062 |
| 14 | *rs879922* | 0.044/0.389 | *G/C* | 0.279 | 0.422 | 0.999 | 0.405 |

*MAF: minor allele frequency; CHB：Han Chinese in Beijing, China；CEU：Utah residents with Northern and Western

European ancestry

# *P*_HWE_ value for non-diabetic (control) participants

Additional Table S3 Baseline characteristics of study participants

|  | **Non-diabetic** | **Diabetic** | **P-value** |
| --- | --- | --- | --- |
| Sample(N) | 272 | 275 | — |
| Male：Female | 110:162 | 95:180 | 0.154 |
| Age(Y) | 58.1±13.0 | 59.2±10.5 | 0.248 |
| Smoking (%) | 55(20.2) | 68(24.7) | 0.207 |
| Drinking (%) | 38(14.0) | 51(18.5) | 0.147 |
| SBP(mmHg) | 115.7±9.5 | 124.2±11.5 | <0.001 |
| DBP(mmHg) | 75.5±8.3 | 81.2±9.6 | <0.001 |
| BMI(Kg/m^2^) | 25.7±4.1 | 28.0±3.8 | <0.001 |
| TRIG (mmol/L) | 1.33±0.49 | 1.63±0.51 | <0.001 |
| CHOL (mmol/L) | 4.74±0.90 | 4.87±1.20 | 0.129 |
| HDL-C(mmol/L) | 1.23±0.17 | 1.18±0.15 | <0.001 |
| LDL-C(mmol/L) | 2.80±0.61 | 2.88±0.64 | 0.124 |
| Lp-A(g/L) | 0.24±0.14 | 0.20±0.13 | 0.001 |
| ApoA1/ApoB | 1.39±0.26 | 1.41±0.35 | 0.281 |
| FBG(mmol/L) | 5.57±0.41 | 6.83±0.71 | <0.001 |
| HbA1C(%) | 5.59±0.59 | 6.57±0.84 | <0.001 |
| Cr(μmol/L) | 69.9±23.6 | 67.3±22.4 | 0.195 |
| BUN(mmol/L) | 5.13±1.77 | 5.14±1.78 | 0.977 |
| UA(μmol/L) | 270.4±91.1 | 328.9±86.5 | <0.001 |
| ALT(U/L) | 25.0±13.3 | 23.2±12.0 | 0.111 |
| AST(U/L) | 24.4±10.9 | 24.1±9.5 | 0.687 |
| Alb(g/L) | 39.5±3.3 | 39.5±8.7 | 0.939 |
| Na^+^(mmol/L) | 139.1±3.6 | 140.4±5.6 | 0.001 |
| K^+^(mmol/L) | 4.23±0.29 | 4.16±0.27 | 0.005 |
| HsCRP(mg/L) | 11.6±12.3 | 12.6±17.3 | 0.418 |
| ACE(U/L) | 40.7±10.4 | 41.6±15.1 | 0.421 |
| Renin(pg/mL) | 32.9±24.3 | 45.8±20.8 | <0.001 |
| Ang I(ng/L) | 2.44±0.85 | 2.48±1.07 | 0.004 |
| Ang II(ng/L) | 122.5±74.5 | 148.9±52.6 | <0.001 |
| ALD(ng/L) | 238.9±79.3 | 251.1±109.1 | 0.136 |
| LVMI(g/m^2^) | 87.1±15.4 | 93.7±16.4 | <0.001 |
| LVEF(%) | 62.1±6.3 | 58.1±7.8 | <0.001 |
| ACR(mg/g) | 88.5±24.8 | 95.5±26.7 | 0.002 |
| CAS≥50%(N/%) | 18/6.6 | 59/21.5 | <0.001 |

Additional Table S4 Association of ACE2 SNPs with T2D in study participants

| **ACE2 SNPs** | | **Non-diabetic**  **(N/%)** | **Diabetic**  **(N/%)** | ***χ2*** | ***P*-value** | **OR(95%CI)*** | ***P*-value*** |
| --- | --- | --- | --- | --- | --- | --- | --- |
| *rs2235306* | *CC+CT* | 76(27.9) | 85(30.9) | 0.580 | 0.446 | 1.42(0.96-2.12) | 0.083 |
|  | *TT* | 196(72.1) | 190(69.1) |  |  | 1.00 |  |
| *rs2285666* | *CC* | 122(44.9) | 12(44.7) | 0.001 | 0.976 | 1.14(0.79-1.63) | 0.482 |
|  | *TT+CT* | 150(55.1) | 152(55.3) |  |  | 1.00 |  |
| *rs4646142* | *CC+CG* | 150(55.1) | 156(56.7) | 0.139 | 0.710 | 0.99(0.69-1.42) | 0.948 |
|  | *GG* | 122(44.9) | 119(43.3) |  |  | 1.00 |  |
| *rs4646142* | *CC* | 84(30.9) | 64(23.3) | 4.012 | 0.045 | 1.00 |  |
|  | *GG+CG* | 188(69.1) | 211(76.7) |  |  | 1.32(0.88-1.99) | 0.182 |
| *rs4646155* | *CC* | 266(97.8) | 251(91.3) | 11.219 | 0.001 | - | - |
|  | *TT* | 6(2.2) | 24(8.7) |  |  | - | - |
| *rs4830542* | *CC+CT* | 108(39.7) | 120(43.6) | 0.869 | 0.351 | 1.00 |  |
|  | *TT* | 164(60.3) | 155(56.4) |  |  | 0.96(0.67-1.39) | 0.847 |
| *rs6632677* | *CC+CG* | 22(8.1) | 30(10.9) | 1.265 | 0.261 | 1.00 |  |
|  | *GG* | 250(91.9) | 245(89.1) |  |  | 0.74(0.40-1.36) | 0.324 |

* After adjustment for nationality, gender, age, smoking and BMI.

Additional Table S5 Association of T2D risk related ACE2 SNPs with elevated blood pressure in study participants

| **ACE2 SNPs** | | **SBP** | | | |  | **DBP** | | | |
| --- | --- | --- | --- | --- | --- | --- | --- | --- | --- | --- |
|  |  | **SBP<130mmHg (N/%)** | **SBP ≥130mmHg**  **(N/%)** | **OR(95%CI)*** | ***P*-value*** |  | **DBP<80mmHg (N/%)** | **DBP ≥80mmHg**  **(N/%)** | **OR(95%CI)*** | ***P*-value*** |
| *rs1978124* | *CC* | 269(64.0) | 62(48.8) | 1.00 |  |  | 177(62.7) | 154(58.6) | 1.00 |  |
|  | *TT+CT* | 151(36.0) | 65(51.2) | 1.47(0.93-2.33) | 0.100 |  | 107(37.7) | 109(41.4) | 0.89(0.61-1.31) | 0.570 |
| *rs2048683* | *GG* | 320(76.2) | 73(57.5) | 1.00 |  |  | 212(74.6) | 181(68.8) | 1.00 |  |
|  | *TT+GT* | 100(23.8) | 54(42.6) | 1.71(1.05-2.78) | 0.032 |  | 72(25.4) | 82(31.2) | 1.11(0.73-1.68) | 0.630 |
| *rs2074192* | *CC* | 181(43.1) | 59(46.5) | 0.67(0.40-1.12) | 0.124 |  | 131(46.1) | 109(41.4) | 1.00 |  |
|  | *TT+CT* | 239(56.9) | 68(53.5) | 1.00 |  |  | 153(53.9) | 154(58.6) | 1.95(1.29-2.93) | 0.001 |
| *rs233575* | *CC+CT* | 108(25.7) | 62(48.8) | 2.32(1.42-3.77) | 0.001 |  | 84(29.6) | 86(32.7) | 0.99(0.66-1.49) | 0.955 |
|  | *TT* | 312(74.3) | 65(51.2) | 1.00 |  |  | 200(70.4) | 177(67.3) | 1.00 |  |
| *rs4240157* | *CC+CT* | 118(21.8) | 70(55.1) | 2.27(1.44-3.57) | <0.001 |  | 90(31.7) | 98(37.3) | 0.97(0.66-1.42) | 0.856 |
|  | *TT* | 302(71.9) | 57(44.9) | 1.00 |  |  | 194(68.3) | 165(62.7) | 1.00 |  |
| *rs4646156* | *AA+AT* | 104(24.8) | 54(42.5) | 1.68(1.03-2.72) | 0.037 |  | 76(26.8) | 82(31.2) | 1.04(0.69-1.56) | 0.863 |
|  | *TT* | 316(75.2) | 73(57.5) | 1.00 |  |  | 208(73.2) | 181(68.8) | 1.00 |  |
| *rs4646188* | *CC+CT* | 124(29.5) | 42(33.1) | 2.05(1.23-3.41) | 0.006 |  | 80(28.2) | 86(32.7) | 1.85(1.22-2.79) | 0.004 |
|  | *TT* | 296(70.5) | 85(66.9) | 1.00 |  |  | 204(71.8) | 177(67.3) | 1.00 |  |
| *rs879922* | *CC+CG* | 202(48.1) | 102(80.3) | 3.46(1.83-6.56) | <0.001 |  | 132(46.5) | 172(65.4) | 2.44(1.60-3.71) | <0.001 |
|  | *GG* | 218(51.9) | 25(19.7) | 1.00 |  |  | 152(53.5) | 91(34.6) | 1.00 |  |

* After adjustment for gender, age, smoking, BMI and T2D.

Additional Table S6 Association of ACE2 SNPs with increased TRIG (≥1.7mmol/L) in study participants

| **ACE2 SNPs** | | **TRIG<1.7mmol/L (N/%)** | **TRIG ≥1.7mmol/L**  **(N/%)** | **OR(95%CI)*** | ***P*-value*** |
| --- | --- | --- | --- | --- | --- |
| *rs1978124* | *CC* | 231(61.8) | 100(57.8) | 1.00 |  |
|  | *TT+CT* | 143(38.2) | 73(42..2) | 0.96(0.64-1.42) | 0.826 |
| *rs2048683* | *GG* | 277(74.1) | 116(67.1) | 1.00 |  |
|  | *TT+GT* | 97(25.9) | 57(32.9) | 1.09(0.71-1.66) | 0.699 |
| *rs2074192* | *CC* | 167(44.7) | 73(42.2) | 0.76(0.50-1.15) | 0.194 |
|  | *TT+CT* | 207(55.3) | 100(57.8) | 1.00 |  |
| *rs233575* | *CC+CT* | 105(28.1) | 65(37.6) | 1.29(0.85-1.96) | 0.224 |
|  | *TT* | 269(71.9) | 108(62.4) | 1.00 |  |
| *rs4240157* | *CC+CT* | 113(30.2) | 75(43.4) | 1.76(1.13-2.75) | 0.013 |
|  | *TT* | 261(69.8) | 98(56.6) | 1.00 |  |
| *rs4646156* | *AA+AT* | 101(27.0) | 57(32.9) | 1.03(0.68-1.57) | 0.893 |
|  | *TT* | 273(73.0) | 116(67.1) | 1.00 |  |
| *rs4646188* | *CC+CT* | 112(29.9) | 54(31.2) | 1.00 |  |
|  | *TT* | 262(70.1) | 119(68.8) | 0.81(0.54-1.22) | 0.323 |
| *rs879922* | *CC+CG* | 187(50.0) | 117(67.6) | 2.21(1.38-3.54) | 0.001 |
|  | *GG* | 187(50.0) | 56(32.4) | 1.00 |  |
| *rs2285666* | *CC* | 154(41.2) | 91(52.6) | 1.71(1.17-2.52) | 0.006 |
|  | *CC+TT* | 220(58.8) | 82(47.4) | 1.00 |  |
| *rs4646142* | *CC+CG* | 219(58.6) | 87(50.3) | 1.00 |  |
|  | *GG* | 155(41.4) | 86(47.4) | 1.54(1.05-2.26) | 0.026 |

* After adjustment for gender, age, BMI and T2D.

Additional Table S7 Association of ACE2 SNPs with decreased HDL-C (<1.0mmol/L) in study participants

| **ACE2 SNPs** | | **HDL-C≥1.0mmol/L (N/%)** | **HDL-C<1.0mmol/L**  **(N/%)** | **OR(95%CI)*** | ***P*-value*** |
| --- | --- | --- | --- | --- | --- |
| *rs1978124* | *CC* | 202(61.2) | 129(59.4) | 1.00 |  |
|  | *TT+CT* | 128(38.8) | 88(40.6) | 1.24(0.85-1.80) | 0.275 |
| *rs2048683* | *GG* | 249(75.5) | 144(66.4) | 1.00 |  |
|  | *TT+GT* | 81(24.5) | 73(33.6) | 2.02(1.33-3.06) | 0.001 |
| *rs2074192* | *CC* | 120(36.4) | 120(55.3) | 1.94(1.33-2.84) | 0.001 |
|  | *TT+CT* | 210(63.6) | 97(44.7) | 1.00 |  |
| *rs233575* | *CC+CT* | 83(25.2) | 87(40.1) | 3.06(2.01-4.67) | <0.001 |
|  | *TT* | 247(74.8) | 130(59.9) | 1.00 |  |
| *rs4240157* | *CC+CT* | 94(28.5) | 94(43.3) | 2.13(1.46-3.13) | <0.001 |
|  | *TT* | 236(71.5) | 123(56.7) | 1.00 |  |
| *rs4646156* | *AA+AT* | 94(28.5) | 94(43.3) | 1.82(1.19-2.77) | 0.006 |
|  | *TT* | 236(71.5) | 123(56.7) | 1.00 |  |
| *rs4646188* | *CC+CT* | 97(29.4) | 69(31.8) | 1.00 |  |
|  | *TT* | 233(70.6) | 148(68.2) | 0.84(0.58-1.24) | 0.389 |
| *rs879922* | *CC+CG* | 166(50.3) | 138(63.6) | 2.76(1.79-4.27) | <0.001 |
|  | *GG* | 164(49.7) | 79(36.4) | 1.00 |  |
| *rs2285666* | *CC* | 136(41.2) | 109(50.2) | 1.89(1.26-2.85) | 0.002 |
|  | *CC+TT* | 194(58.8) | 108(49.8) | 1.00 |  |

* After adjustment for gender, age, BMI and T2D.

Additional Table S8 Association of T2D risk related ACE2 SNPs with increased LDL-C (≥1.8mmol/L) in study participants

| **ACE2 SNPs** | | **LDL-C<1.8mmol/L (N/%)** | **LDL-C ≥1.8mmol/L**  **(N/%)** | **OR(95%CI)*** | ***P*-value*** |
| --- | --- | --- | --- | --- | --- |
| *rs1978124* | *CC* | 108(65.5) | 223(58.4) | 1.00 |  |
|  | *TT+CT* | 57(34.5) | 159(41.6) | 1.17(0.77-1.77) | 0.471 |
| *rs2048683* | *GG* | 129(78.2) | 264(69.1) | 1.00 |  |
|  | *TT+GT* | 36(21.8) | 118(30.9) | 1.76(1.10-2.80) | 0.018 |
| *rs2074192* | *CC* | 79(47.9) | 161(42.1) | 0.88(0.58-1.33) | 0.544 |
|  | *TT+CT* | 86(52.1) | 221(57.9) | 1.00 |  |
| *rs233575* | *CC+CT* | 50(30.3) | 120(31.4) | 1.11(0.72-1.71) | 0.641 |
|  | *TT* | 115(69.7) | 262(68.6) | 1.00 |  |
| *rs4240157* | *CC+CT* | 58(35.2) | 130(34.0) | 0.92(0.61-1.39) | 0.692 |
|  | *TT* | 107(64.8) | 252(66.0) | 1.00 |  |
| *rs4646156* | *AA+AT* | 36(21.8) | 122(31.9) | 2.64(1.49-4.68) | 0.001 |
|  | *TT* | 129(78.2) | 260(68.1) | 1.00 |  |
| *rs4646188* | *CC+CT* | 39(23.6) | 127(33.2) | 1.00 |  |
|  | *TT* | 126(76.4) | 255(66.8) | 0.64(0.42-1.01) | 0.054 |
| *rs879922* | *CC+CG* | 70(42.4) | 234(61.3) | 1.89(1.24-2.88) | 0.003 |
|  | *GG* | 95(57.6) | 148(38.7) | 1.00 |  |

* After adjustment for gender, age, BMI and T2D.

Additional Table S9 Association of T2D risk related ACE2 SNPs with increased CHOL (≥5.2mmol/L) in study participants

| **ACE2 SNPs** | | **CHOL<5.2mmol/L (N/%)** | **CHOL ≥5.2mmol/L**  **(N/%)** | **OR(95%CI)*** | ***P*-value*** |
| --- | --- | --- | --- | --- | --- |
| *rs1978124* | *CC* | 252(59.0) | 79(65.8) | 1.97(1.23-3.14) | 0.005 |
|  | *TT+CT* | 175(41.0) | 41(34.2) | 1.00 |  |
| *rs2048683* | *GG* | 306(71.7) | 87(72.5) | 2.34(1.31-4.18) | 0.004 |
|  | *TT+GT* | 121(28.3) | 33(27.5) | 1.00 |  |
| *rs2074192* | *CC* | 188(44.0) | 52(43.3) | 0.70(0.43-1.14) | 0.147 |
|  | *TT+CT* | 239(56.0) | 68(56.7) | 1.00 |  |
| *rs233575* | *CC+CT* | 129(30.2) | 41(34.2) | 0.90(0.55-1.45) | 0.653 |
|  | *TT* | 298(69.8) | 79(65.8) | 1.00 |  |
| *rs4240157* | *CC+CT* | 133(31.1) | 55(45.8) | 1.34(0.86-2.09) | 0.201 |
|  | *TT* | 294(68.9) | 65(54.2) | 1.00 |  |
| *rs4646156* | *AA+AT* | 125(29.3) | 33(27.5) | 1.00 |  |
|  | *TT* | 302(70.7) | 87(72.5) | 1.89(1.12-3.21) | 0.018 |
| *rs4646188* | *CC+CT* | 141(33.0) | 25(20.8) | 1.00 |  |
|  | *TT* | 286(67.0) | 95(79.2) | 1.59(0.96-2.65) | 0.072 |
| *rs879922* | *CC+CG* | 217(50.8) | 87(72.5) | 2.26(1.35-3.79) | 0.002 |
|  | *GG* | 210(49.2) | 33(27.5) | 1.00 |  |

* After adjustment for gender, age, BMI and T2D.

Additional Table S10 The HbA1C level difference between different genotypes of T2D risk related ACE2 SNPs in study participants with or without T2D

| **ACE2 SNPs** | | HbA1C (%) | | |
| --- | --- | --- | --- | --- |
|  |  | **Non-diabetic** | **Diabetic** | ***P-value*** |
| *rs1978124* | *CC* | 5.43±0.63 | 6.46±0.80 | <0.001 |
|  | *TT+CT* | 5.45±0.48 | 6.67±0.87 | <0.001 |
|  | *P-value* | 0.781 | 0.041 |  |
| *rs2048683* | *GG* | 5.43±0.60 | 6.50±0.80 | <0.001 |
|  | *TT+GT* | 5.45±0.55 | 6.67±0.90 | <0.001 |
|  | *P-value* | 0.881 | 0.112 |  |
| *rs2074192* | *CC* | 5.49±0.53 | 6.74±0.63 | <0.001 |
|  | *TT+CT* | 5.41±0.62 | 6.40±0.98 | <0.001 |
|  | *P-value* | 0.279 | 0.001 |  |
| *rs233575* | *CC+CT* | 5.32±0.58 | 6.65±0.86 | <0.001 |
|  | *TT* | 5.47±0.59 | 6.51±0.83 | <0.001 |
|  | *P-value* | 0.093 | 0.189 |  |
| *rs4240157* | *CC+CT* | 5.36±0.57 | 6.80±0.91 | <0.001 |
|  | *TT* | 5.46±0.59 | 6.38±0.73 | <0.001 |
|  | *P-value* | 0.230 | <0.001 |  |
| *rs4646156* | *AA+AT* | 5.44±0.53 | 6.74±0.90 | <0.001 |
|  | *TT* | 5.44±0.60 | 6.50±0.80 | <0.001 |
|  | *P-value* | 0.972 | 0.112 |  |
| *rs4646188* | *CC+CT* | 5.43±0.56 | 6.67±0.72 | <0.001 |
|  | *TT* | 5.44±0.61 | 6.54±0.88 | <0.001 |
|  | *P-value* | 0.873 | 0.273 |  |
| *rs879922* | *CC+CG* | 5.45±0.54 | 6.76±0.86 | <0.001 |
|  | *GG* | 5.43±0.62 | 6.23±0.69 | <0.001 |
|  | *P-value* | 0.796 | <0.001 |  |

**References**

1. Meschia JF, Klaas JP, Brown RD, Jr., Brott TG: Evaluation and Management of Atherosclerotic Carotid Stenosis. Mayo Clinic proceedings 2017, 92(7):1144-1157.

2. Lang RM, Badano LP, Mor-Avi V, Afilalo J, Armstrong A, Ernande L, Flachskampf FA, Foster E, Goldstein SA, Kuznetsova T et al: Recommendations for cardiac chamber quantification by echocardiography in adults: an update from the American Society of Echocardiography and the European Association of Cardiovascular Imaging. European heart journal cardiovascular Imaging 2015, 16(3):233-270.
